# SWOT analysis of risk factors associated with introduction of African Swine Fever through vehicles returning after export of pigs

**DOI:** 10.3389/fvets.2022.1049940

**Published:** 2023-01-04

**Authors:** Yuqi Gao, Lisbeth Harm Nielsen, Anette Ella Boklund, Mart C. M. de Jong, Lis Alban

**Affiliations:** ^1^Quantitative Veterinary Epidemiology, Department of Animal Sciences, Wageningen University and Research, Wageningen, Netherlands; ^2^Department for Food Safety and Veterinary Issues, Danish Agriculture and Food Council, Copenhagen, Denmark; ^3^Department of Veterinary and Animal Sciences, Faculty of Health and Medical Sciences, University of Copenhagen, Copenhagen, Denmark

**Keywords:** qualitative analysis, risk assessment, disease introduction, ASF, Denmark

## Abstract

Denmark is a major pig exporter and applies a high level of biosecurity, with washing and disinfecting stations for returning livestock vehicles. The introduction of African Swine Fever (ASF) would have significant economic consequences related to loss of export of live pigs and products thereof. In this study, we focused on the role of empty livestock vehicles returning after exports of pigs for the introduction of ASF. Initially, the current components and measures related to export of livestock were described. Next, analyses of strengths, weaknesses, opportunities, and threats (SWOT) were conducted, covering the components and measures identified. Then, export of pigs was described either through assembly centers or directly from farms. Washing and disinfection, as required and undertaken at the designated stations, constitutes the most important among all risk-reducing measures identified. Recommendations are to: (1) ensure the quality of washing and disinfection through staff training; (2) find new, safe, and more efficient disinfectants; (3) ensure the required temperature, and therefore effect, of the disinfectant and water. It was impossible to assess, the influence of export through assembly centers compared to direct transport. However, through SWOT analyses we identified the strengths and weaknesses of the two pathways. Moreover, components/measures with risks of unknown sizes are also discussed, such as vehicles undertaking cabotage and the current vehicle quarantine periods.

## 1. Introduction

Denmark, mostly surrounded by sea, has only 68 km of land border with Germany, which eases the ability to establish high levels of biosecurity at the borders to protect against introduction of exotic livestock hazards. Denmark is one of the largest pig exporters in Europe (1). In 2021, Denmark exported 14.5 million live pigs, whereas another 17.4 million finishing pigs were slaughtered inside the country. Moreover, 79% (1,597,359/2,029,000 tons) of the pig meat produced was exported (2). Therefore, the introduction of a notifiable disease in pigs would have huge economic consequences due to loss of export of live pigs and pig meat (3).

African Swine Fever (ASF) constitutes a threat to the global pig industry. ASF, caused by African Swine Fever Virus (ASFV), is not zoonotic but can be transmitted between domestic pigs and wild boars of all ages. Despite the transmission rate for ASFV is lower than observed for Classical Swine Fever Virus and Foot and Mouth Disease Virus (4, 5), ASFV is still infamous because of its high mortality rate (6, 7), multiple transmission routes including direct and indirect contact (8), long-term viability in the environment due to persistence of the virus in various materials and animal tissues (9–12), lack of effective vaccines (13), and last but not least, huge economic consequences. Since the outbreak of ASF genotype II in Georgia in 2007, the area in Europe affected by this genotype has gradually expanded. The main markets for Danish pig exports are Poland, with an ongoing epidemic since 2014, and Germany, which has been affected since September 2020 and has registered seven domestic outbreaks of ASF since the beginning of 2021. Therefore, precautionary measures related to export of pigs are very important for the Danish pig industry and the Danish Veterinary and Food Administration (DVFA).

There are many potential pathways that could lead to introduction of ASF to a naïve pig population, including import of pigs/pork, human-related activities (swill feeding, visits by veterinarians, hunting tourism, etc.), wild boar movements, and returning livestock vehicles (14, 15). Indirect exposure to contaminated environments was identified by ESFA as a likely route of ASF infection in domestic pigs and wild boar (16). In Denmark, empty contaminated livestock vehicles returning after export and not well-cleaned and disinfected are considered one of the main risks (17), because: (1) annual pig imports into Denmark are low, e.g., 47 pigs were imported in 2017–2021; (2) large numbers of vehicles export live pigs, e.g., 26,918 vehicles exported livestock in 2021, among which 25,252 were pig exports (18), and; (3) there are almost no free-living wild boar (19). In 2019, a fence was erected along the border with Germany, reducing the risk of migrating wild boars entering from northern Germany (20). Combined with Denmark's active culling policy on wild boars, the probability of introducing ASF through wild boars is considered very low (21).

In this study, with respect to the risk of introduction of ASF, we focused on the role of empty livestock vehicles returning after export. We firstly examined how the export system is arranged and run. Secondly, we focused on the type of export: (1) directly from pig farms or (2) indirectly *via* assembly centers, as these two options could differ with respect to the probability of introducing ASF into Denmark. The aims were to:

- Analyze the strengths, weaknesses, opportunities, and threats (SWOT) of all components and measures identified in the Danish pig transportation system.
- Compare the risk of ASF introduction through returning vehicles exporting pigs from assembly centers and directly from farms.

An additional aim was to identify which more detailed studies to initiate. The outcome of the study targets public and private risk managers, in and outside Denmark, who are interested in effective measures to reduce the risk of introducing ASF *via* returning livestock vehicles.

## 2. Materials and methods

### 2.1. Data collection

Between November and December 2021, two of us (YG & LN) visited one organic, two conventional pig farms, and two assembly centers. These farms and assembly centers were selected based on their representation of the main types of farms in Denmark (Farm size, export size, geographical location, partnerships, etc.). During the farm visits, the entire structure of the farm was inspected from the perspective of external biosecurity. Focus was on entrance of humans and pigs including the delivery facilities. The owner and the daily manager participated in these visits and discussions were taken about the procedures in place on the farm. Additionally, all three existing washing and disinfecting stations approved by the Danish Agriculture and Food Council (DAFC) were visited. Here, the author LN is the expert, which facilitated the systematic inspection from the arrival of a vehicle at the station until its departure after washing and disinfection. Relevant details were inspected such as measures in place to ensure the required temperature of the disinfectant agent and the photo control of each vehicle to document compliance with the rules. This was followed by a visit to two assembly centers located in two different parts of the country. Again, a systematic inspection was undertaken, following the pigs as they moved from the Danish side to the export side. The owner and the daily manager participated in the discussions taking place during the visits. Focus was on how cross-contamination could happen between vehicles and people in the assembly centers. Moreover, published reports and scientific papers, expert opinions, and various statistics were obtained. Finally, information was retrieved about the three private standards applied to Danish pig production [DANISH Product Standard (22), DANISH Transport Standard (23), and Global Red Meat Standard (24)].

### 2.2. SWOT analysis

SWOT analysis is qualitative, fact-based, structured, and provides realistic descriptions of business planning and functioning (25). SWOT analysis was performed to elucidate the roles of key components and measures in the Danish pig export transportation system to identify their strengths (S), weaknesses (W), opportunities (O), and threats (T). Here, S and O refer to factors that could be helpful in achieving the purpose, and W and T refer to those that could be obstacles to achieving the purpose. From the analytical source perspective, S and W can be considered as having internal origins and O and T as having exogenous origins (26). SWOT analysis was chosen, because it is an adequate tool for how to develop comprehensive and suitable strategies based on the reality of the situation.

The SWOT analysis was conducted by all authors, and the results were subsequently discussed and updated separately with representatives from the pig industry and the Danish Veterinary and Food Administration (DVFA) in two rounds, with preliminary discussions in December 2021, followed by final discussions in May 2022.

### 2.3. Comparison of two different routes of pig export

Danish pig producers can either export their pigs directly from the farm or move the pigs to an assembly center from where they are exported. The purpose of assembly centers is to separate export vehicles from vehicles used nationally. To illustrate the differences in these processes, two simplified mappings were constructed to enable comparison of the risk of ASFV introduction. The comparison utilized data from the DAFC, the current Danish standards, and the SWOT analysis.

## 3. Results and discussion

### 3.1. Description of the Danish pig export system

#### 3.1.1. Danish pig herds

Between 2011 and 2021, the number of Danish pig farms decreased from 9,069 to 8,117 (2). Specialization is increasing, so e.g., some farms only have sows, which produce piglets up to 7 or 30 kg, whereas other farms specialize in buying either 7 or 30 kg piglets and raise these to the finisher stage. Moreover, the number of outdoor farms is increasing. By 24 August 2022, there were 468 farms with outdoor pigs, including organic farms and farms holding fenced-in wild boars, whereas in 2011, there were 314 outdoor pig farms. Furthermore, there are hobby farms with pigs and farms with pet pigs. Danish legislation states outdoor farms must be entirely double-fenced. In 2021, 68% of Danish pig farms, covering 97% of pigs produced, were part of the DANISH Product Standard, implying the farms comply with housing and management rules (27). Moreover, most sow farms are specific pathogen free (SPF) farms, so comply with external biosecurity requirements. Under this, the herd's health status is monitored routinely for the presence of infections, such as *Mycoplasma* lung disease, *Actinobacillus pleuropneumoniae*, swine dysentery, Porcine Reproductive and Respiratory Syndrome (PRRS) virus, atrophic rhinitis, scabies, and lice. According to SPF-Sund, 78% of all pigs born in Denmark are from a sow herd with SPF status (28).

Livestock vehicles enter the pig farm area to load or unload pigs for breeding, raising, slaughter, or export. Most farms have a special area for loading that is separated from the other farm facilities, and the area is cleaned and disinfected after use. Such safe delivery facilities are highly recommended by the Danish SPF system. If this is not established, the pig producers are advised to place the pigs in a trailer that is moved away from the farm before the pigs are loaded onto the livestock vehicle.

#### 3.1.2. EU requirements for livestock movement

In accordance with European Union (EU) Regulation (29), all livestock vehicles should be cleaned and disinfected immediately after every transport of animals. This is conducive to the prevention and control of infectious diseases. However, the legislation does not require control of the vehicles regarding the quality of the washing and disinfection (30). The DVFA studied the effectiveness of washing and disinfection for Danish pig export vehicles. In 2018, 42% of the vehicles were inadequately cleaned and disinfected; this reduced in 2020–2021 to 15% of vehicles being unsatisfactorily cleaned and disinfected (31, 32).

Cabotage road transport constitutes another potential source of contamination by pigs that may result in increased risk of ASF introduction. Cabotage means that vehicle drivers have the right to carry out three transport services within the EU Member State the vehicle has gone to (33). Although the livestock vehicle registers the countries to which pig export is destined, there is a lack of knowledge in the Danish system, regarding additional destinations, because the TRACES system is set up to only share information about movements from one's own country.

#### 3.1.3. Washing and disinfecting stations

In addition to the washing and disinfection required by the EU after unloading, three washing and disinfecting stations have been set up in Denmark. Two are located in the western part of the country, i.e., southern Jutland close to the Danish/German border, whereas one is located in the eastern part of the country, close to a ferry with a connection to Germany. These privately-run stations are financed and supervised by DAFC.

A series of washing and disinfection procedures have been set up based upon the following considerations: a room temperature of 20°C and disinfection solution temperatures of 20–25°C are enquired to ensure effectiveness. The vehicles to be treated are not necessarily washed with soap. Therefore, organic material may be present before disinfection takes place. This needs to be considered when assessing the effect of the disinfection product. When washing at the DANISH approved cleaning and disinfecting sites, the contact time is short - around 10 min - and disinfection may take place on partially wet surfaces, which may limit the effect of disinfection. The ambient temperature on the vehicles is low during a large part of the year, again potentially lowering the effect.

Moreover, the product is used in closed indoor environments, where plenty of staff is working at all times of the day. Therefore, the product must comply with the Danish Working Environment Regulation, which prescribes use of products that are non-harmful for humans and the environment. The following issues must be complied with before a disinfection product will be approved by the DAFC: Data from laboratory tests must be provided because scientific articles are not accepted as documentation. Such laboratory tests may be performed at accredited laboratories. Test results must be provided, which should show correlation between concentration, contact time and temperature relating to African Swine Fever, Foot-and-Mouth Disease, and Classical Swine Fever. The laboratory tests must be performed at different temperatures down to 5°C. Moreover, the product safety data sheet must be provided. Finally, documentation must be provided that the product is registered on the list of relevant substances and the respective substance and product suppliers, in accordance with Article 95 of the EU Biocidal Products Regulation No. 528/212.

For example, glutaraldehyde is a high-level disinfectant, but it cannot be used for the disinfection of vehicles. Because according to the safety datasheet for glutaraldehyde (g5882, sigmaaldrich.com), this substance is harmful if inhaled or swallowed and toxic by inhalation, skin contact and ingestion. In general, there are only three disinfection products left to use in the DANISH system: Virkon S, Kiemkill, and Vanodox.

All vehicles in the DANISH Transport Standard system are required to be washed and disinfected after entering Denmark, and as stated above, the service is free of charge for farmers and transport companies. According to DAFC, there is full compliance with the rules regarding washing and disinfection of all vehicles. Washing certificates are issued based on, among other things, the vehicle's GPS data. Data covering 4 weeks in each of spring and autumn, 2021, showed that around 40% of export vehicle drivers upload their vehicle's GPS data to DAFC's webserver (Unpublished data from DAFC). The remaining drivers could have various reasons for not uploading GPS data—see below.

#### 3.1.4. ASF risk zones

Risk zones regarding ASF are defined by DAFC based on evaluations covering outbreak conditions, proximity to outbreak zones, and ocean currents. The risk zones are updated whenever the epidemiological situation changes, and new risk zones are placed on the DAFC website (23). The risks related to neighboring countries are described by colors; black, red, and green, in decreasing order of risk. The color of the zone, from which a vehicle returns, determines the type of certificate and the quarantine policy required for the vehicle. Black certificates are issued for vehicles unable to or uninterested in submitting GPS data, and for vehicles returning from black zones. If a vehicle has been in a black zone, a 7-day quarantine is imposed before a new transport can be done directly from farms. In contrast, green certificates impose the minimum 2-day quarantine when exporting directly from a farm. Quarantine rules are explained in detail on DAFC's website (23). The type of certificate is considered by drivers when they plan their next transport: in-country transport, export transport *via* an assembly center, or export transport with direct access to a Danish farm.

#### 3.1.5. Assembly centers

Altogether, 29 DANISH-approved, privately owned assembly centers operate in Denmark. The services in these centers are paid for by the exporter. Arriving vehicles are foreign vehicles or Danish export vehicles. Each center is divided into two sides: a Danish side, open only to vehicles arriving from Danish farms to unload pigs, and an export side, open only to vehicles arriving to load pigs. The middle part of the center has tunnels that connect the two sides and several pig pens. The pigs to be exported are inspected by official veterinarians, focusing on health conditions as part of the fit-for-transport assessment, undertaken as the pigs pass through a tunnel. To limit the spread of infection, tunnels are used in one direction only, so pigs go from the Danish side to the export side. Moving pigs from an assembly center instead of directly from a Danish pig farm is preferred by most vehicle drivers with black certificates to avoid the 7-day quarantine period.

### 3.2. SWOT analyses

The results of SWOT analyses of the washing and disinfecting stations, quarantine period, ASF risk zone identification, and cabotage driving are shown (Tables 1, 2). SWOT analyses for the two different routes of exporting pigs from Denmark are shown (Table 3).

**Table 1:** SWOT analysis of components and measures at washing and disinfecting stations for returning livestock vehicles.

**Washing and disinfecting stations for livestock vehicles returning from outside Denmark**	
**Strengths (S)**	**Weaknesses (W)**
The use of the washing and disinfecting stations is required by the Danish livestock industry, effectively implying that all livestock vehicles must be washed and disinfected when entering Denmark Three washing stations are available, and use is free of charge implying that returning vehicles (and utensils) are cleaned and disinfected outside and inside At an initial 100% visual check, vehicles with visible dirt are sent back to further cleaning and disinfection. The vehicle is cleaned on the outside, followed by disinfection in- and outside for at least 20 min with disinfectant kept at 25°C, along with random bacteriological sampling ≥ disinfection effect is secured. In 2021 a total number of 3,523 so-called Hygicult E/β – GUR samples were taken of 543 different vehicles. This random test is an indicator of how effective the disinfection is at the DANISH approved washing and disinfecting stations.	The washing effect greatly depends on the washing staff. In rare cases, visible dirt can still be found after washing and disinfection Boots in the cabin are inspected but not washed, and the vehicle cabin is not inspected or washed The use of the washing and disinfecting stations is required by the Danish livestock industry, but it is not a legal requirement, and it costs a lot of money to run these facilities. The results of samples taken from the vehicles after washing and disinfection show variation. A preliminary analysis of these data indicate that a substantial part of the variation may be related to the cleaning status of the vehicle when it arrives in Denmark
**Opportunities (O)**	**Threats (T)**
The current system creates awareness among vehicle drivers regarding the importance of having clean vehicles entering Denmark New disinfectants, which are more efficient than the currently used, which meet the safety standards of the working environment of staff, could be identified	The low temperature in winter can cool the disinfectant, reducing its effectiveness and allowing ASFV to remain viable After crossing the border, the vehicle can go elsewhere before going to the washing station The waste water arising after washing is not allowed to be reused, but the current disinfection process and effectiveness of wastewater treatment are unknown
**Recommendations**	
1. Provide station staff with continued education to maintain their understanding of the importance of washing and disinfection 2. Improve temperature control of both disinfectant and water, especially in cold seasons 3. Investigate new disinfection systems regarding safety, effect and costs	
**Quarantine period for livestock vehicles**	
**Strengths (S)**	**Weaknesses (W)**
The quarantine system encourages vehicles from risky zones to load pigs at assembly centers, likely reducing the number of such vehicles entering Danish farms Weekly tracking of vehicle compliance with quarantine periods is performed by DAFC	ASFV is very persistent in the environment especially at low temperature; ASFV's viability in the current quarantine periods during cold months is considered inadequate
**Opportunities (O)**	**Threats (T)**
In warmer months (>20°C), a quarantine period shorter than 7-day is sufficient, even if the vehicle was contaminated and not effectively cleaned and disinfected	Where and how vehicles spend the quarantine period is unknown and uncontrolled. If a dirty vehicle is close to an outdoor farm, indirect spread could occur
**Recommendation**	
1. Use GPS data from all vehicles for the last 7 days before entering Denmark to classify vehicles more correctly according to risk zones	
**Identification of ASF risk zones**	
**Strengths (S)**	**Weaknesses (W)**
Denmark identifies, and updates weekly, ASFV risk zones wider than those published by EUROSTAT and OIE Washing certificates are mainly based on the risk zones, which are strictly distinguished. When the vehicle provides a complete GPS record covering the last 7 days, an appropriate certificate is issued. Otherwise, a black certificate is issued	Green zones in the DANISH Transport Standard could contain undetected ASF-infected pigs/wild boars, especially if translocations over long distances occur. The risk zone classification is not fully evidence-based, but is a management tool where confidence in the veterinary system is included in a non-specific way
**Opportunities (O)**	**Threats (T)**
Pig producers need only check the vehicle's washing certificate, which is clear and straightforward	Domestic transport vehicles as part of cabotage do not require a washing certificate from a DANISH washing and disinfecting station, and so are a risk for ASFV spread
**Recommendation**	
1. Identify and implement timely information collection regarding new ASF outbreaks	

**Table 2:** SWOT analysis of livestock vehicles used for cabotage.

**National transport/cabotage**	
**Strengths (S)**	**Weaknesses (W)**
National transport/cabotage results in efficient use of vehicles, possibly enabling cost-effective transportation	A returned vehicle, after washing, disinfection, and quarantine, can move pigs inside Denmark. If ASFV in a contaminated vehicle remains viable during these procedures, the virus could spread to pigs being moved from one Danish farm to another
**Opportunities (O)**	**Threats (T)**
Carrying out national transport/cabotage requires additional quarantine time for vehicles coming from risky zones, which will reduce the number of vehicles undertaking national transport/cabotage, thereby reducing the risk	National transport/cabotage in areas with detected/undetected ASF is riskier than similar transports within Denmark. Pigs from potentially infected zones could be loaded onto vehicles. Once a vehicle is contaminated, ASFV could be introduced to many pig farms before the first case is detected
**Recommendation**	
1. Run awareness campaigns regarding the importance of vehicle washing and disinfection 2. Open the TRACES system to share information about the movements of all pigs, because the TRACES system is currently set up to only share information about movements from one's own country	

**Table 3:** SWOT analysis of two methods for exporting pigs from Denmark; through assembly centers or directly from farms.

**Export through assembly centers**	
**Strengths (S)**	**Weaknesses (W)**
Centers have a Danish side and an export side, reducing the probability of domestic vehicles coming into contact with vehicles from high-risk zones Vehicles returning from outside Denmark do not come in close proximity to Danish farms	Drivers, official veterinarians, and staff can walk back and forth between Danish and export sides, facilitating cross-contamination The centers' pens, aisles, and tunnels are only washed intermittently, and not between all batches of pigs
**Opportunities (O)**	**Threats (T)**
It may be easier to prevent introduction of ASFV at a low number pf assembly centers compared to at a high number of pig farms undertaking direct export	If the vehicle is not or only ineffectively washed and disinfected Multiple vehicles congregate at the assembly centers simultaneously. ASFV from one vehicle could spread to other vehicles ASFV can enter the center if vehicles are insufficiently washed and disinfected
**Recommendations**	
1. Implement continued education for assembly center staff and other persons on preventing ASFV 2. Instigate random controls of cleanliness and procedures	
**Export through direct transport from farms**	
**Strengths (S)**	**Weaknesses (W)**
Pigs stay in the same vehicle during the whole export process, reducing the risk of contracting other infections A minority of the vehicles have a high level of biosecurity, e.g., SPF vehicles for export of breeding pigs. These vehicles have air filters to prevent airborne transmission of pig pathogens	Vehicles returning from ASF risk zones can go to Danish pig farms after having been cleaned, disinfected, and quarantined. If the regulations regarding washing, disinfection, and quarantine are not followed, a risk of ASFV spread could occur Recommendations regarding pig loading are not always followed. Occasionally: (i) drivers enter the inside of the farm area; (ii) employees leave the farm area, and; (iii) the delivery area is not fully washed and disinfected after loading
**Opportunities (O)**	**Threats (T)**
Pig producers could be more aware of ASF prevention measures	ASFV can be introduced to Danish farms if: (i) If the vehicle is not or only ineffectively washed and disinfected; (ii) quarantine rules are not followed, and; (iii) farm delivery facilities are inadequate
**Recommendation**	
1. Instigate continued education for pig producers using direct export regarding preventing ASF and other hazards every time a vehicle arrives 2. Increase the frequency of random controls of cleanliness	

Based on the results of the SWOT analysis, the washing and disinfection conducted at the three stations seem to constitute the most effective way of preventing introduction of ASF compared to the other measures identified (quarantine period, assembly center/direct to farm, and cabotage/national transport). This is because washing and disinfection takes place at the first stop after the vehicle enters Denmark. Moreover, there is some uncertainty regarding the appropriate length of the quarantine period in the cold months.

In summary, the recommendations that seem most feasible are related to the washing and disinfection. ASFV, as a complex enveloped virus, is susceptible to detergents such as soaps, as well as to several disinfectants and dehydration (34). However, many disinfectants are unsuitable for use in practice, because of safety issues: the cleaning staff are using disinfectants in a confined space and, therefore, any disinfectant that may cause skin or eye irritation or be suspect of carcinogenic effect is not permitted to be used in Denmark. This rules out e.g., glutaraldehyde. Many factors need to be taken into account, when setting up a robust system, e.g., if organic material is present when disinfected, the efficiency of disinfectants like chlorine compounds and oxidizing agents will be reduced (34). The search for new, safe and more efficient disinfectants that can be applied is important. But for the present, focus should be on how to ensure properly performed cleaning and disinfection, using the required temperature of the disinfectant and water. This involves staff training, where a future study of knowledge, attitudes and practices (KAP) may add valuable information to further ensure effectiveness of the system in place.

### 3.3. Comparison of different routes of exporting pigs

**Figure 1:**
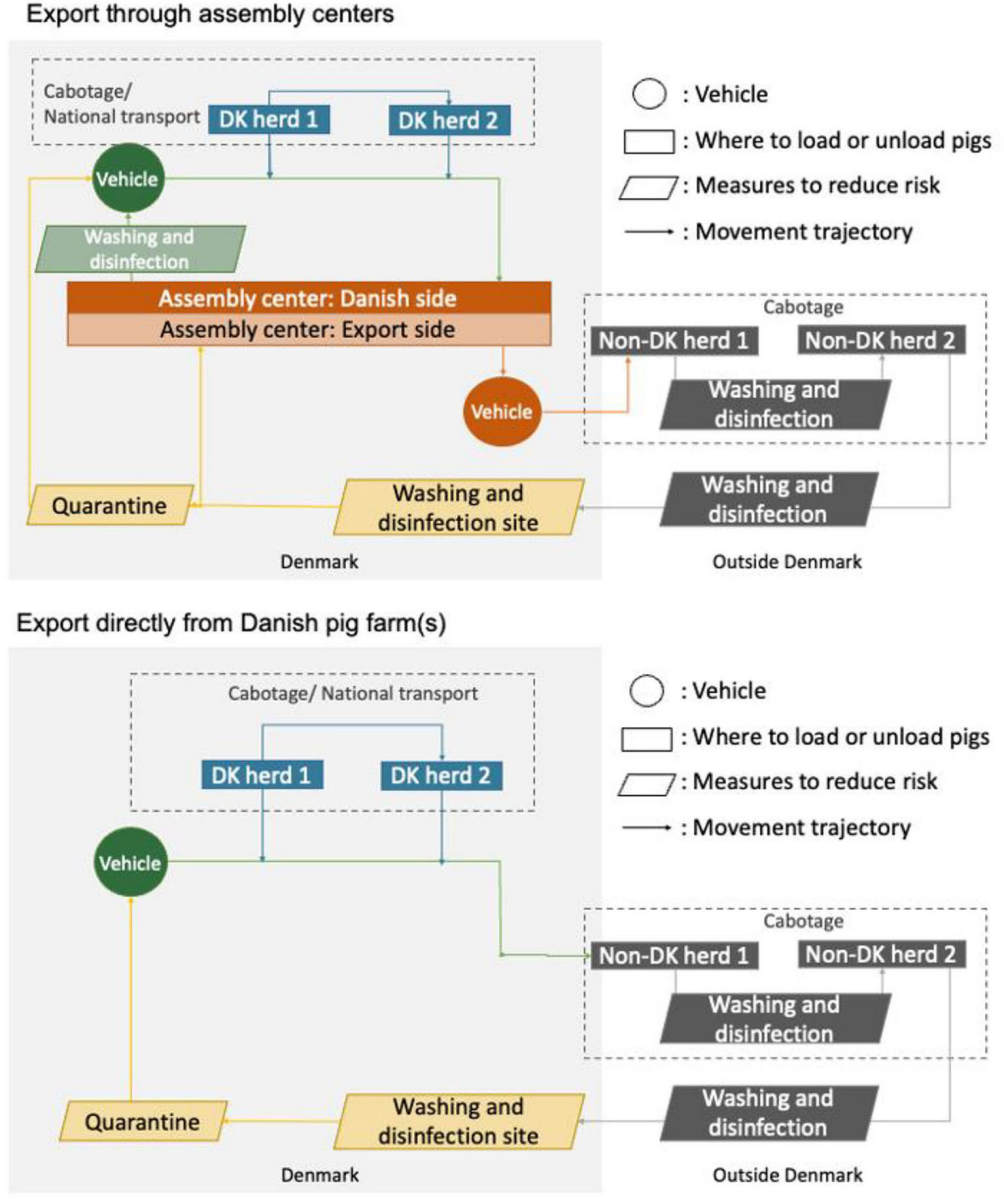
Simplified mappings of the Danish pig transportation system. The **upper** panel depicts pig export through assembly centers, while the **lower** panel depicts pig export directly from Danish pig farm(s).

In 2021, about 60% of annual pig exports were *via* assembly centers, while 40% were exported directly from farms. When the vehicle is outside Denmark, there is no difference between the two export methods (Figure 1). Differences lie in two aspects. (1) Before export, the vehicle can load pigs directly from one or more farms and head directly to the receiving farm located in another country. Alternatively, the vehicle with pigs from a Danish farm can head to an assembly center and unload all pigs for veterinary control. Thereafter, the pigs are reloaded in the same or another vehicle. (2) Vehicles returning to Denmark are assigned differing quarantine periods, e.g., if a vehicle with a black certificate is scheduled to enter a Danish farm, a 7-day quarantine period is applied, but no quarantine is required if the vehicle goes to an assembly center. See detailed explanation on the DAFC website (https://pigresearchcentre.dk/DANISH-quality-assurance-scheme/The-Danish-Transport-Standard).

For direct export, we found the 7-day quarantine period is sufficient for ASFV, which naturally decays when the temperature is above 20°C. This is supported by Olesen et al. (35), reporting short ASFV viability times in non-cleaned experimental facilities at 20°C. However, our preliminary results show ASFV can remain viable longer at lower temperatures (36). Hence, the 7-day quarantine period before visiting a Danish pig farm could be insufficient especially when the temperature is lower than 10°C. However, the increased risk of longer ASFV viability time in cold months will also greatly increase the risk related to assembly centers, and therefore, the comparison of risk between the two routes of exporting pigs is difficult and deserves further attention.

Still, export *via* assembly centers is considered by the Danish pig production sector to be associated with a high level of prevention of introduction of ASFV into Denmark, because foreign vehicles do not have contact with Danish pig farms. However, we were unable to assess the relative risk related to export through assembly centers compared to direct export from farms. More research is needed, e.g., assessing the risk of cross-contamination between vehicles and people in the assembly centers. In line, the environmental transmission rates in the assembly center are unknown, and this area deserves further attention. A next step could be to undertake a KAP study among the persons involved in the different areas of the system, to understand in more details, where the limitations are: in the knowledge of the persons working in the system, their attitudes, or the practices which result from the system.

Pig producers who prefer to export directly from their farm are advised to ensure that each incoming vehicle is clean and has a valid washing certificate, implying the driver has complied with vehicle quarantine rules. Moreover, proper use of safe delivery facilities is recommended to prevent hazards on/in vehicles from entering farms. However, more knowledge is needed regarding compliance with these recommendations.

In view of these considerations, this study recommends the continuation of washing and disinfection at the assembly centers, as this is an important activity preventing ASFV from entering Denmark, as stated by Bronsvoort et al. (17), who also pointed to issues regarding the quality of washing and disinfecting transport vehicles.

## 4. Conclusion

This study characterized the current components and measures related to export of pigs from Denmark. The SWOT analysis contributed to better understanding of maintaining a low probability of introducing ASF. Denmark already has a high level of biosecurity preparedness. However, there are some areas that might constitute potential risks.

The main recommendations concern washing and disinfecting undertaken at the designated three stations. Focus should be on continuously ensuring the effect of washing and disinfection, which is of paramount importance, particularly in cold months, when the 7-day quarantine is likely insufficient for ASFV to decay enough to avoid transmission. This involves offering staff training and controlling sufficiently high temperatures of wash water and disinfectant during the year, in particular from September to March, to ensure the efficacy of washing and the correct application of the disinfectants. Moreover, DAFC should search for new, safe and more effective disinfectants.

We were unable to assess relative risks of export from assembly centers compared to direct export from farms. Further research is needed, including a KAP study among personnel in assembly centers as well as related to direct transport. Both export routes have their advantages and disadvantages. Hence, pig farmers and other persons involved need to follow best practices when applying any of the two ways of exporting.

## Data availability statement

The original contributions presented in the study are included in the article/supplementary material, further inquiries can be directed to the corresponding author.

## Author contributions

LA, MJ, LN, and AB contributed to the conception and design of the study. LN and YG carried out the farm visits and collection of information. LA, LN, AB, and YG brainstormed during the SWOT analysis and wrote parts of the manuscript. YG wrote the first draft of the manuscript. All authors participated in the revision of the manuscript, read, and approved the submitted version.
